# The tyrosine kinase inhibitor nilotinib targets the discoidin domain receptor DDR2 in calcific aortic valve stenosis

**DOI:** 10.1111/bph.15911

**Published:** 2022-07-19

**Authors:** Miguel Carracedo, Sven‐Christian Pawelzik, Gonzalo Artiach, Marianne G. Pouwer, Oscar Plunde, Peter Saliba‐Gustafsson, Ewa Ehrenborg, Per Eriksson, Elsbet Pieterman, Leif Stenke, Hans M. G. Princen, Anders Franco‐Cereceda, Magnus Bäck

**Affiliations:** ^1^ Department of Medicine Solna Karolinska Institutet Stockholm Sweden; ^2^ Department of Cardiology Karolinska University Hospital Stockholm Sweden; ^3^ Metabolic Health Research, Gaubius Laboratory The Netherlands Organization of Applied Scientific Research (TNO) Leiden The Netherlands; ^4^ Theme Cancer, Division of Hematology Karolinska University Hospital Stockholm Sweden; ^5^ Department of Molecular Medicine and Surgery Karolinska Institutet Stockholm Sweden

**Keywords:** cardiovascular disease, chronic myeloid leukaemia, tyrosine kinase inhibitors, valvular heart disease

## Abstract

**Background and Purpose:**

Tyrosine kinase inhibitors (TKI) used to treat chronic myeloid leukaemia (CML) have been associated with cardiovascular side effects, including reports of calcific aortic valve stenosis. The aim of this study was to establish the effects of first and second generation TKIs in aortic valve stenosis and to determine the associated molecular mechanisms.

**Experimental Approach:**

Hyperlipidemic APOE*3Leiden.CETP transgenic mice were treated with nilotinib, imatinib or vehicle. Human valvular interstitial cells (VICs) were isolated and studied in vitro. Gene expression analysis was perfromed in aortic valves from 64 patients undergoing aortic valve replacement surgery.

**Key Results:**

Nilotinib increased murine aortic valve thickness. Nilotinib, but not imatinib, promoted calcification and osteogenic activation and decreased autophagy in human VICs. Differential tyrosine kinase expression was detected between healthy and calcified valve tissue. Transcriptomic target identification revealed that the discoidin domain receptor DDR2, which is preferentially inhibited by nilotinib, was predominantly expressed in human aortic valves but markedly downregulated in calcified valve tissue. Nilotinib and selective DDR2 targeting in VICs induced a similar osteogenic activation, which was blunted by increasing the DDR2 ligand, collagen.

**Conclusions and Implications:**

These findings suggest that inhibition of DDR2 by nilotinib promoted aortic valve thickening and VIC calcification, with possible translational implications for cardiovascular surveillance and possible personalized medicine in CML patients.

## Graphical abstract

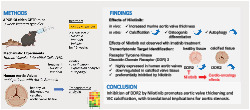

**Abbreviations**
BMP: bone morphogenetic protein
CAVS: calcific aortic valve stenosis
CKD: chronic kidney disease
CML: chronic myeloid leukaemia
ECM: extracellular matrix
Runx2: runt‐related transcription factor 2
TKI: tyrosine‐kinase inhibitors
VIC: valvular interstitial cell
VSMC: vascular smooth muscle cell

**What is already known?**

- Cardio‐oncology involves cardiotoxic effects of chemotherapies as well as increased cardiovascular risk.

**What does this study add?**

- Nilotinib, used to treat chronic myeloid leukaemia (CML), caused aortic valve thickening and calcification.

**What is the clinical significance?**

- This study raises awareness leading to earlier diagnosis and treatment of valve disease in CML.

## 1 INTRODUCTION

Cardio‐oncology has raised the concern that beneficial pharmacological effects in cancer in parallel may be deleterious for the cardiovascular system. In chronic myeloid leukaemia (CML), the Bcr‐Abl tyrosine‐kinase inhibitors (TKI) dramatically improve CML prognosis at the price of cardiovascular adverse events. The use of second generation TKI in CML (Dahlen et al., 2016; Herrmann, 2016) is associated with peripheral arterial occlusive disease and myocardial infarction. This has led to the introduction of cardiovascular risk monitoring and prevention in haematological treatment recommendations for CML (Steegmann et al., 2016).

Recently, rapid progression of calcific aortic valve stenosis (CAVS) under nilotinib treatment was reported in CML (Carracedo et al., 2020). Calcification and thickening of the aortic valve, accompanied by inflammation (Aikawa et al., 2007), extracellular matrix (ECM) remodelling (Artiach, Carracedo, Seime, et al., 2020), and inftraleaflet haemorrhages (Laguna‐Fernandez et al., 2016), are part of the underlying pathological processes of CAVS, which eventually reduce the aortic valve opening causing left ventricular outflow obstruction and heart failure. Several contributors to CAVS incidence and progression have been identified, including traditional cardiovascular risk factors—dyslipidaemia (Smith et al., 2014), obesity (Larsson, Wolk, Hakansson, & Back, 2017), and smoking (Larsson, Wolk, & Bäck, 2017)—genetic predisposition (Larsson et al., 2020; Mathieu et al., 2017; Plunde et al., 2020), and comorbidities in terms of diabetes mellitus (Larsson et al., 2018) and chronic kidney disease (CKD) (Vavilis et al., 2019). In addition, direct exposure to ionizing radiation during radiotherapy increases aortic valve calcification and CAVS (Donnellan et al., 2017). However, the effects of targeted cancer therapies, such as TKIs, on valvular heart disease have remained elusive.

Based on the recent observation of a possible link between nilotinib and CAVS in CML (Carracedo et al., 2020), the aim of the present study was to establish the effects of first‐ and second‐generation TKIs on pathophysiological aortic valve processes. First, we examined in vivo effects in a murine CAVS model. Second, we performed a target screen for nilotinib by transcriptomic analysis of human aortic valves. Third, we addressed molecular mechanisms for TKI‐induced effects in human valvular interstitial cells (VICs), by addressing osteogenic differentiation and calcification as well as effects on cell viability, which can act as a link between pharmacological anti‐tumour effects and cardiovascular side effects. In particular, the TKI nilotinib is a known modulator of autophagy (Kumar et al., 2021), which is a protective mechanism in CAVS (Carracedo, Persson, et al., 2019). The results identified the discoidin domain receptor DDR2, a target of the TKI nilotinib, which was further examined as an interacting point with aortic valve collagen and for associations with clinical CAVS characteristics.

## 2 METHODS

### 2.1 Animals

Animal studies are reported in compliance with the ARRIVE guidelines (Percie du Sert et al., 2020) and with the recommendations made by the *British Journal of Pharmacology* (Lilley et al., 2020). Female APOE*3Leiden.CETP transgenic mice (9 to 14 weeks of age) from the SPF breeding stock at TNO‐Metabolic Health Research (TNO‐Leiden, The Netherlands) were used in this study. Animal experiments were approved by the Institutional Animal Care and Use Committee of the Netherlands Organization for Applied Research under registration number 3557. During the study, mice were housed under standard conditions with a 12‐h light–dark cycle and had free access to food and water. Body weight, food intake, and clinical signs of behaviour were monitored regularly during the study. Mice were fed a semisynthetic diet containing saturated fat from 15% (w/w) cacao butter and 0.15% cholesterol (Western‐type diet; Hope Farms, Woerden, The Netherlands). Pharmacokinetic studies were performed as described previously (Pouwer et al., 2018). After a run‐in period of 3 weeks on WTD, mice were matched in four equal groups based on body weight, age, plasma cholesterol and triglycerides followed by randomization to treatments. Based on the results of a previous pharmacokinetic study, mice received a once daily oral gavage with nilotinib (10 or 30 mg·kg^−1^) or a twice daily gavage with imatinib (150 mg·kg^−1^). The TKIs were suspended in 5% carboxymethyl cellulose. The control mice received a twice daily gavage with the vehicle (5% carboxymethyl cellulose), and the mice receiving a once daily gavage with nilotinib received a second gavage with the vehicle alone. The TKIs were purchased at LC laboratories (Woburn, MA, USA). After 16 weeks of treatment, all animals were killed by CO_2_ inhalation, and death was confirmed by exsanguination.

### 2.2 Patients

Human aortic valves were obtained from patients undergoing aortic valve replacement surgery. The study was approved by the local ethics committee Regionala etikprövningsnämnden i Stockholm (2012/1633‐31/4) and in agreement with the Declaration of Helsinki. All patients gave informed consent.

### 2.3 Human aortic valve preparation, RNA extraction, and gene expression analysis

For RNA extraction, 64 aortic valves were immersed in RNA Later (Qiagen, Hilden, Germany) immediately after surgical removal and stored at 4°C until transported to the laboratory. Macroscopic dissection was performed in the RNA Later solution, dividing each valve into healthy, intermediate, or calcified regions as previously described (Artiach, Carracedo, Plunde, et al., 2020; Laguna‐Fernandez et al., 2016; Nagy et al., 2011). In brief, macroscopically transparent and pliable valvular tissue was defined as healthy, while nonpliable and nontransparent tissue was termed calcified. Thickened samples that were pliable but not transparent were termed intermediate.

Whenever possible, one piece was used for histological analysis, and the adjacent one was frozen at −80°C for RNA extraction.

Genome‐wide expression data were obtained for all three tissue types from each valve by using the Affymetrix Human Transcriptome Array 2.0 as previously described (Sarajlic et al., 2021). The Affymetrix Transcriptome Analysis Console (TAC) 4.0.2 (Thermo Fisher Scientific, Waltham, MA, USA) was used to adjust between arrays that were run in different batches, and data were normalized with signal space transformation‐robust multichip analysis, yielding log2‐transformed expression values.

### 2.4 Cell stimulation

Primary VIC cultures were seeded at a density of 10^4^ cells·cm^−2^ in culture medium with 5% FBS and stimulated with DMSO, nilotinib, imatinib, 7rh (all from Sigma Aldrich, St. Louis, MO, USA), or WRG‐28 (ProbeChem Biochemicals, Shaghai, China). VICs were cultured in polystyrene plates (TPP) for all experiments except when cultured in collagen type 1‐coated plates (Falcon). In vitro *c*alcification was induced by culturing VIC for 9 days in cell culture media supplemented with 2.6‐mM inorganic phosphate (Sigma), prepared as previously described (Carracedo, Artiach, et al., 2019; Carracedo, Witasp, et al., 2019). For autophagy determination, VICs were stimulated for 2 h with bafilomycin A1 (100nM) and thoroughly washed with PBS before further treatments (Carracedo, Persson, et al., 2019; Saliba‐Gustafsson et al., 2019). Viability was assessed after 24 h by WST‐1 reagent (Roche) according to manufacturer's protocol (Carracedo, Persson, et al., 2019).

### 2.5 Aortic valve histology

Hearts from *n* = 15 control mice, *n* = 10 nilotinib (10 mg·kg^−1^)‐treated mice, *n* = 13 nilotinib (30 mg·kg^−1^)‐treated mice, and *n* = 14 imatinib (150 mg·kg^−1^)‐treated mice were isolated, fixed in formalin, and embedded in paraffin and serially sectioned from the proximal 1 mm of the aortic root. Sections were collected upon first aortic valve appearance. Serial cross sections (5 μm thick with intervals of 50 μm) were taken and mounted on AAS‐coated slides. These sections were stained with haematoxylin‐phloxine‐saffron (HPS) for aortic valve thickness and area evaluation. Thickness of the aortic valve was measured on every level at the nodule of Arantius of each leaflet (Simolin et al., 2009). In addition, aortic valve area was measured also on every level in the three leaflets. Aortic valve thickness and area per mouse were defined by the average of the thickness and area at each leaflet, respectively. Quantification was performed using the software Aperio ImageCope (Leica Biosystems Imaging, version: 12.3.3). Immunohistochemistry was performed in conformity with *BJP* Guidelines (Alexander et al., 2018) in murine aortic valve sections using anti‐mouse MAC‐3 (1:50; BD Pharmingen, Netherlands, RRID:AB_393587) and anti‐mouse BMP2 (1:200; Invitrogen, Waltham, MA, USA; RRID:AB_2064111). For total collagen determination, sections were stained with Picro Sirius Red. For iron determination, sections were stained with Prussian Blue Stain according to manufacturer's protocol (Abcam). Colour intensity was determined in ImageJ (version: 1.52a). All quantifications were performed blindly.

### 2.6 VIC isolation and culture

Immediately after surgical removal, human aortic valves were immersed in cell culture medium (DMEM, 10% FBS, 100 units·ml^−1^ penicillin, 100 μg·ml^−1^ streptomycin, 1‐mM sodium pyruvate, 10‐mM HEPES, and 2‐mM L‐glutamine) at 4°C and were transported to the laboratory. Complete valves containing different disease stages were digested using collagenase I and dispase II for 16 h as previously described (Artiach, Carracedo, Plunde, et al., 2020; Laguna‐Fernandez et al., 2016). VICs were seeded onto tissue culture polystyrene containers, and culture medium was exchanged every other day. Cells were used for experiments between passages 1 and 3. Cell culture reagents were purchased from Gibco; plastics were from Corning.

### 2.7 Calcification quantification

In vitro calcification was assessed in VICs after 9 days in culture in medium supplemented with 2.6‐mM phosphate. Calcification was quantified by extracting the calcium‐phosphate salts with 0.6‐N HCl and quantified using a calcium colorimetric assay (Sigma‐Aldrich, MAK022) according to manufacturer's protocol.

### 2.8 TaqMan real‐time PCR

Reverse‐transcription was performed using High Capacity RNA‐to‐cDNA Kit (Thermo Fisher Scientific, Waltham, MA, USA). Quantitative real‐time PCR reaction was developed on a 7900HT Fast Real‐Time PCR system (Life Technologies) using Taqman Assay‐on‐Demand from Life Technologies. The relative mRNA expression of the target genes was quantified by the 2^−**Δ**CT^ or 2^−**ΔΔ**CT^ method using TATA binding protein (TBP) as endogenous control.

### 2.9 RNA extraction and quality assessment

Total RNA from tissue and cells in culture was isolated using the RNeasy Lipid Tissue Mini kit (Qiagen). RNA concentrations were quantitated spectrophotometrically at 260 nm (Thermo Scientific), and quality was evaluated on a 2100 Bioanalyzer (Agilent) using RNA 6000 NANO chips to assess the RNA integrity number.

### 2.10 Immunoblotting

For protein analysis, human aortic valves were collected in phenol red‐free DMEM supplemented with 10% fetal calf serum (FBS), dissected and stored at −80°C. Western blotting was performed in conformity with BJP Guidelines. For bone morphogenetic protein (BMP)‐2 immunoblotting, protein extracts were prepared by lysing cultured VICs with RIPA buffer (Sigma Aldrich, St. Louis, MO, USA) containing a protease and phosphatase inhibitor cocktail (Roche, Basel, Switzerland). Protein concentrations were determined using DC‐Protein Assay (BioRad, Hercules, CA, USA) following manufacturer's protocol; 15 μg of the protein extracts were diluted (1:1) in Laemmli sample buffer (BioRad, Hercules, CA, USA) containing 2.5% β‐mercaptoethanol (Sigma Aldrich). Diluted samples were incubated 5 min at 95°C, loaded on a MiniProtean TGX 4–12% gel (BioRad), and finally transferred to a PVDF membrane (BioRad). The membranes were blocked using 5% BSA and incubated with primary antibodies against BMP‐2 (ThermoFisher, 710022, RRID:AB_2532532) and the loading control vinculin (Abcam, Cambridge, UK; ab207440) overnight at 4°C. Membranes were then washed and incubated in fluorescently labelled antibodies (Li‐Cor, Lincoln, NE, USA) for 1 h at room temperature, and signal was detected using an Odyssey CLx imagin system (Li‐Cor). BMP‐1 levels were normalized to vinculin to control for unwanted sources of variation.

For microtubule‐associated protein 1A/1B‐light chain 3 (LC‐3) immunoblotting, protein extracts were loaded on a 14% acrylamide SDS‐PAGE gel and transferred to PVDF membranes (BioRad). The membranes were blocked using 5% milk and incubated with primary antibodies against LC‐3 (Novus Biologicals, Littleton, CO, USA; NB100‐2220; RRID:AB_10003146). A HRP‐conjugated secondary antibody (BioRad) was used to amplify the signal. Blots were developed using an enhanced chemiluminescence reagent kit (GE Healthcare, Chicago, IL, USA). ImageJ was used for densitometry, and autophagy flux was defined as LC3‐II fold‐change between experimental conditions supplemented with bafilomycin A1 over experimental condition without bafilomycin A1 by using LC‐II density normalized to β‐actin (ThermoFisher, AM4302, RRID:AB_2536382) to control for unwanted sources of variation.

### 2.11 DDR1 and DDR2 ELISA

Protein extracts from ex vivo valves and VICs were prepared using lysis buffer (R&D, DYC002) supplemented with aprotinin and leupeptin (both from Tocris). DDR1 and DDR2 protein was assessed by using the DuoSet IC ELISA against DDR1, p‐DDR1, DDR2, and p‐DDR2 (all from R&D).

### 2.12 Data and analysis

The manuscript complies with *BJP*'s recommendations and requirements on experimental design and analysis (Curtis et al., 2018). Results are expressed as either mean ± S.D. or mean with 95% CI. Results are shown as fold change compared with controls for semiquantitative mRNA and protein analyses of paired experiments to account for unwanted sources of variation, indicated as fold change on the y‐axis. In each experiment, biological repeats were performed to ensure consistent responses in samples from different individuals serving as valve donors for mRNA and protein analyses, as well as sources of VICs used for cell cultures. No exclusion of outliers was performed in the data analysis of the present study. Sample size was based on previous studies of valve thickness in murine CAVS models (Artiach, Carracedo, Plunde, et al., 2020) and studies to evaluate biological effects in primary cultures of isolated VICs (Artiach, Carracedo, Plunde, et al., 2020; Carracedo et al., 2020; Carracedo, Persson, et al., 2019; Laguna‐Fernandez et al., 2016). When using VICs, each dot represents one individual VIC donor. Differences in *n* in in vitro experiments are due to availability of patient VIC donor cells at the time of the experiment.

Statistical significance of differences between groups of at least *n* = 5 for normally distributed data was assessed with Student's *t* test when comparing two groups and with one‐way or two‐way ANOVA as appropriate followed by recommended post hoc tests for multiple comparisons only if *F* was significant and there was no variance inhomogeneity. For non‐normally distributed data, statistical significance was assessed using the Friedman test followed by a Dunn's multiple comparison test when appropriate. Associations were established using Spearman correlations. Statistical significance was assigned at *P* < 0.05, and statistical difference levels were assigned as follows in the Figures, *P < 0.05. Statistical analysis was performed using GraphPad Prism 7 (GraphPad Software Inc, CA, USA).

### 2.13 Nomenclature of targets and ligands

Key protein targets and ligands in this article are hyperlinked to corresponding entries in http://www.guidetopharmacology.org and are permanently archived in the Concise Guide to PHARMACOLOGY 2021/22 (Alexander et al., 2021).

## 3 RESULTS

### 3.1 Nilotinib increases aortic valve thickness in mice

**FIGURE 1:**
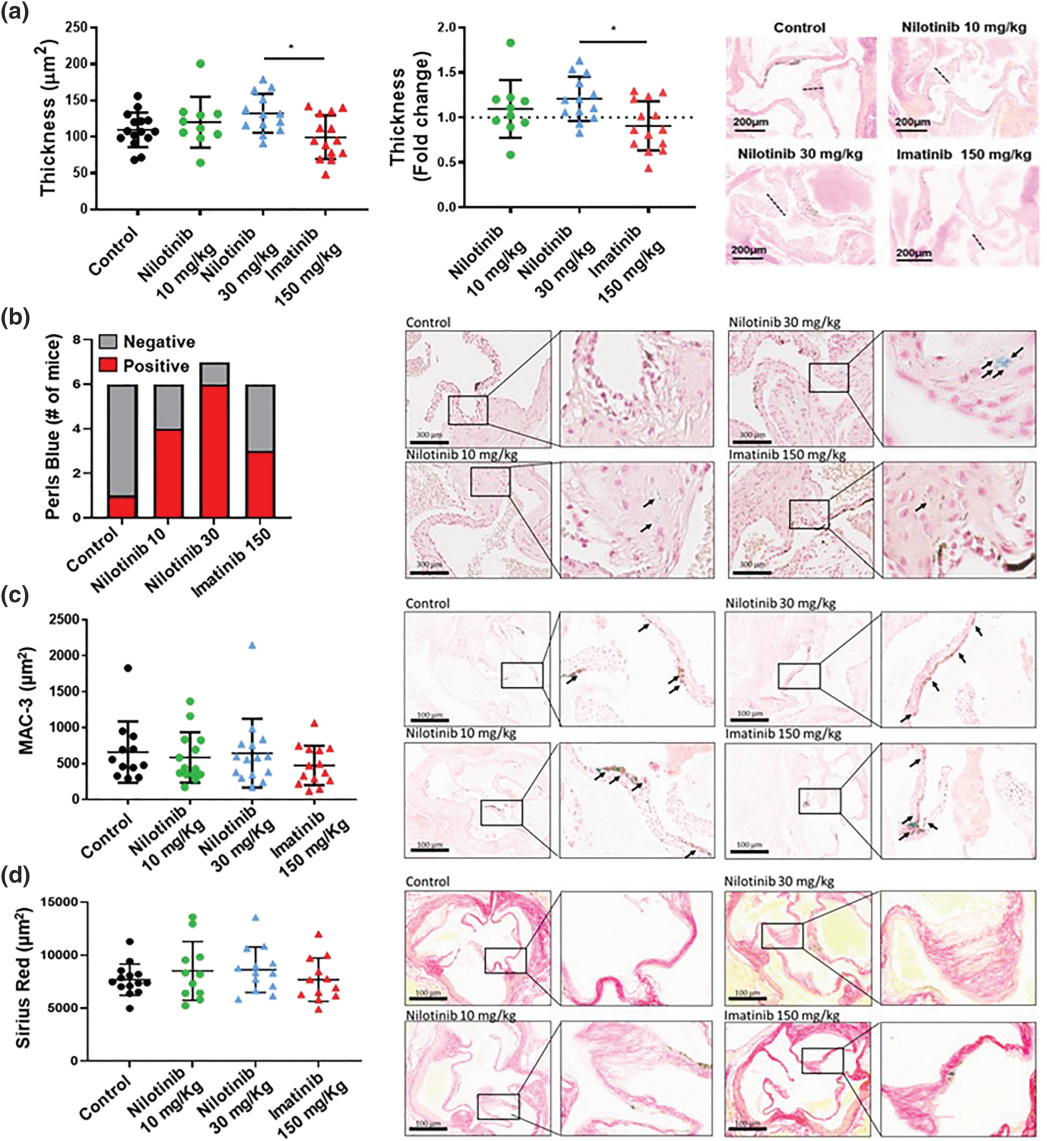
Effects of nilotinib on murine aortic valves. (a) Quantification of aortic valve thickness measured at the nodule of Arantius in micrometre, and expressed as fold change to control. *n* = 15 vehicle treated mice (dotted line), *n* = 10 nilotinib (green circles) 10 mg·kg^−1^, *n* = 13 nilotinib 30 mg·kg^−1^ (blue squares) and *n* = 14 imatinib 150 mg·kg^−1^ (red triangles). *P < 0.05 for one‐way ANOVA. Right panel shows representative images of the aortic valve in HPS stained sections. (b) Perls blue (iron) positivity quantification and representative photomicrographs. DMSO vs. nilotinib 30 mg·kg^−1^; *P* < 0.05 by Fisher's exact test. (c) MAC‐3 (macrophages) immunohistochemistry quantification, and representative photomicrographs. (d) Sirius Red (collagen) histochemistry quantification, and representative photomicrographs. In panels b and c, data are represented as mean ± S.D. *n* = 6 control mice, *n* = 6 nilotinib 10 mg·kg^−1^, *n* = 7 nilotinib 30 mg·kg^−1^, and *n* = 6 imatinib 150 mg·kg^−1^

Hyperlipidaemic APOE*3Leiden.CETP transgenic mice not only develop spontaneous atherosclerosis but can also be used for induction of lesions in the aortic valve with similarities to changes observed in human CAVS (van Broekhoven et al., 2019). After 16 weeks of treatment with nilotinib (30 mg·kg^−1^), APOE*3Leiden.CETP transgenic mice developed a significant 1.2‐fold (95% CI 1.059–1.355) increase in valve thickness compared with control mice, and a similar significant increase compared with the imatinib group (Figure 1a). In contrast, neither a lower dose of nilotinib (10 mg·kg^−1^; 95% CI 0.87–1.3) nor imatinib (150 mg·kg^−1^; 95% CI 0.75–1.1) showed a significant effect as compared with control. Histologically, nilotinib‐treated mice exhibited a higher proportion of iron positivity (Figure 1b), whereas no significant differences in either MAC‐3 (CD107b) positivity (Figure 1c) or Sirius Red staining for collagen content (Figure 1d) were detected between the groups. The osteogenic marker BMP2 was detected in the valve leaflets, with most prominent stainings detected in valve sections derived from nilotinib‐treated mice (Figure S1).

### 3.2 Nilotinib promotes calcification, osteogenic signalling, and inhibits autophagy in human valvular interstitial cells

**FIGURE 2:**
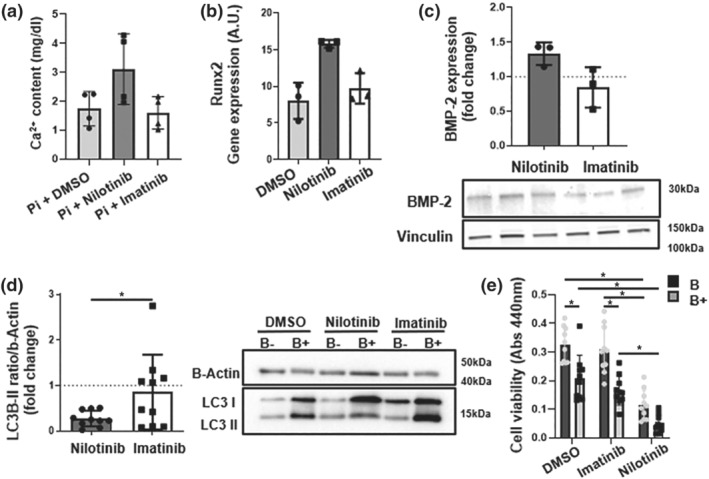
Effects of nilotinib on human valvular interstitial cells. (a) Quantification of phosphate‐induced calcification of human valvular interstitial cells (VICs) after 9 days. (b) mRNA expression of the osteogenic transcription factor RUNx‐2 in VICs treated for 24 h. (c) Immunoblotting against BMP‐2 in human VICs after 24 h treatment with control (DMSO), nilotinib (10 μM), or imatinib (10 μM). (d) Immunoblotting against LC3‐I and LC3‐II in human VICs treated for 2 h with or without bafilomycin (100 nM) followed by 24 h with control (DMSO), nilotinib (10 μM), or imatinib (10 μM). (e) VIC viability after inhibition of autophagy with bafilomycin (B+) (100 nM) or vehicle control (B−) (DMSO) for 2 h, followed by treatment with control (DMSO), nilotinib (10 μM) or imatinib (10 μM) for 24 h. Data are represented as mean ± S.D. Statistical significance of differences between groups were determined when at least *n* = 5 (d and e). *P < 0.05 Student's *t* test. Each dot represents one patient VIC donor

Nilotinib, but not imatinib, increased calcification 1.97 ± 0.06‐fold (95% CI 1.81–2.14) in preliminary findings using VICs (Figure 2a). This was accompanied by increased expression of the osteoblast signature gene runt‐related transcription factor 2 (Runx2; Figure 2b) and an increase in the osteogenic activator BMP‐2 in VICs (Figure 2c). Nilotinib, but not imatinib, significantly inhibited autophagic activity in VICs as measured by LC3 lipidation after bafilomycin treatment (Figure 2d). VIC viability was decreased by nilotinib to the same degree as inhibition of autophagy with bafilomycin (Figure 2e). In addition, bafilomycin did not have an additive effect on nilotinib treatment on VIC viability (Figure 2e).

### 3.3 Niotinib inhibits the tyrosine kinase DDR2 in human aortic valves

**FIGURE 3:**
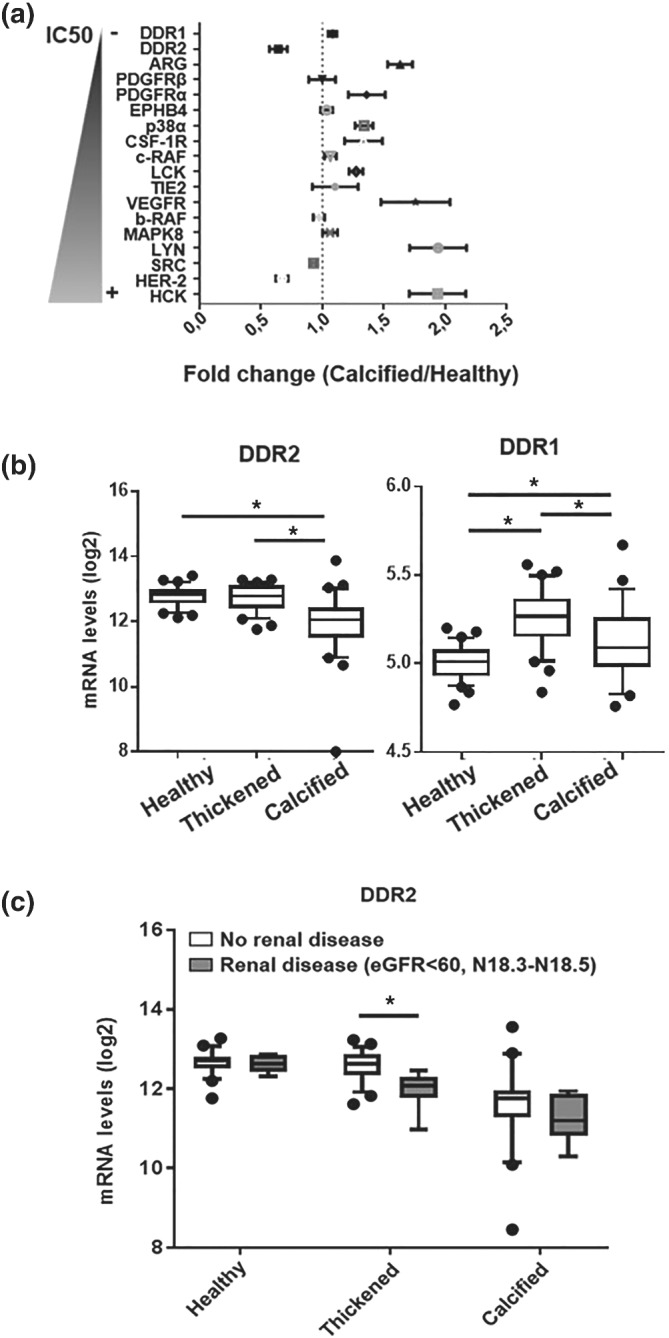
The expression of tyrosine kinases in human aortic valves and its relation to stage of disease and renal function. (a) Tyrosine kinase fold change gene expression between healthy and calcified tissue in human aortic valves ranked by their reported nilotinib IC_50_. Data presented as 95% CI, *n* = 64. (b) mRNA expression of the tyrosine kinases DDR2 and DDR1 in healthy, thickened and calcified tissue of human aortic valves. Data are presented as median and 5–95 percentile, *n* = 64. *P < 0.05, for one‐way ANOVA followed by post hoc test for multiple comparisons (c) DDR2 mRNA expression in healthy, thickened and calcified tissue of human aortic valves stratified according to the absence or presence of renal disease, defined as indicated. No renal disease *n* = 58, renal disease *n* = 6. *P < 0.05 vs. no renal disease. Data are presented as 5–95 percentiles

To determine the potential effectors for the valvular effects of nilotinib, a target screen was performed by transcriptomic analysis of human aortic valves. The expression levels of all tyrosine kinases susceptible to inhibition by nilotinib were mapped in human aortic valves. The CAVS stages are identifiable macroscopically, allowing the identification of healthy, intermediate, and calcified parts of the afflicted cusps in surgically removed stenotic aortic valves (Artiach, Carracedo, Plunde, et al., 2020; Laguna‐Fernandez et al., 2016; Nagy et al., 2011). Figure 3a shows nilotinib‐sensitive tyrosine kinases ranked by their IC_50_ for nilotinib inhibition indicating the fold‐change in expression between calcified and noncalcified tissues derived from the same human aortic valve. Table 1 shows the same tyrosine kinases ranked by their valvular expression levels in the present study. Only three nilotinib‐targeted tyrosine kinases were significantly downregulated in calcified tissue as compared with healthy tissue, namely, DDR2 (95% CI 0.57–0.72), SRC: (95% CI 0.90–0.96), and HER‐2 (95% CI 0.62–0.72) (Figure 3a). Of these, DDR2 is the tyrosine kinase for which nilotinib has the highest affinity (Day et al., 2008) and was the second highest expressed in healthy human aortic valve tissue in this study (Table 1).

**TABLE 1:** Tyrosine kinase expression levels in human aortic valves

Tyrosine kinase	IC50 (nM ± SEM)	Healthy (*N* = 64)	Thickened (*N* = 64)	Calcified (*N* = 64)	P for RM ANOVA ON RANKS
PDGFR alpha	71 ± 7	7656 (2062)	8748 (2102)	9825 (3679)	<0.0001
DDR2	5.2 ± 3.3	7129 (1311)	6970 (1658)	4535 (2198)	<0.0001
PDGFR beta	53 ± 4.6	1962 (780.1)	2071 (890.3)	1781 (681.3)	0.427
b‐RAF	3600 ± 150	802.3 (86.55)	820.2 (92.49)	773.9 (143.7)	0.0232
c‐RAF	1100 ± 270	503.1 (48.59)	522.2 (65.42)	532.4 (88.87)	0.017
TIE2	2800 ± 710	381.3 (328.3)	275.8 (203.3)	280.5 (141.7)	0.014
LYN	4000 ± 860	308.7 (74.88)	342.1 (125.6)	588.9 (296.8)	<0.0001
CSF‐1R	677 ± 437	265.4 (94.98)	251.2 (91.39)	323.8 (135.9)	0.0087
p38α	570 ± 100	244.6 (31)	265.5 (31.37)	324.2 (65.28)	<0.0001
MAPK8	3900 ± 620	166.8 (24.85)	171.2 (25.77)	175.3 (39.4)	0.1607
ARG	<20	157.6 (32.09)	190.7 (50.72)	251.2 (54.83)	<0.0001
HER‐2	7200 ± 1,400	112.1 (23.24)	104.6 (20.79)	72.9 (18.21)	<0.0001
HCK	7500 ± 830	75.76 (20.27)	88.52 (38.08)	144.9 (76.15)	<0.0001
VEGFR	3200 ± 970	53.4 (22.35)	48.8 (17.81)	81.08 (39.72)	<0.0001
EPHB4	250	47.12 (6.832)	45.63 (5.742)	47.88 (6.799)	0.0545
SRC	4600 ± 520	33.97 (2.875)	33.19 (2.615)	31.33 (2.539)	<0.0001
DDR1	3.7 ± 1.2	32.19 (1.895)	38.61 (3.724)	34.82 (4.352)	<0.0001
LCK	1300 ± 430	27.74 (3.457)	28.92 (3.676)	35.02 (5.415)	<0.0001

*Note*: Tyrosine kinases organized by highest to lowest gene expression level in the healthy tissue of the valve. RM‐ANOVA ON RANKS: repeated measures analysis of variance on ranks.

Since the target screen identified DDR2, the second most inhibited tyrosine kinase by nilotinib (Manley et al., 2010), as one of the highest expressed tyrosine kinases in human aortic valves and one of only three significantly downregulated tyrosine kinases in calcified valve tissue, the DDR pathways were further explored. DDR2 was predominantly expressed over DDR1 in human aortic valves, with DDR1 being among the lowest expressed kinases in healthy aortic valve tissue (Table 1). Whereas DDR2 mRNA levels were almost halved in calcified compared with healthy valve tissue, DDR1 mRNA levels significantly increased in thickened and calcified valve tissue (Figure 3b). Protein levels of DDR1 and 2 exhibited similar changes (Figure S2). Aortic valve DDR2 expression was inversely associated with mRNA levels of the osteogenic marker RUNx2, which was significant in intermediate and calcified tissue. Significant positive associations were detected for expression levels of DDR2 with autophagy markers (Table 2). Whereas there was no apparent difference between nilotinib and imatinib for the inhibition of DDR1 phosphorylation, DDR2 phosphorylation was inhibited by nilotinib, but not imatinib (Figure S2), in line with previous results from other assays (Day et al., 2008).

**TABLE 2:** Correlations for DDR2 expression with autophagy markers after stratification into different tissue characteristics of the aortic valve

	Healthy (*N* = 64)		Thickened (*N* = 64)		Calcified (*N* = 64)	
	Rho	*P*	Rho	*P*	Rho	*P*
**RUNx2**	−0.23	0.06	**−0.37**	**0.003**	**−0.44**	**3** ^**x**^ **10** ^**−4**^
**ULK1**	−0.16	0.21	−0.02	0.91	**0.42**	**10** ^**−4**^
**ATG9A**	**−0.32**	**0.01**	−0.05	0.71	**0.40**	**0.001**
**ATG10**	**0.45**	**3** ^**x**^ **10** ^**−4**^	**0.52**	**10** ^**−5**^	**0.59**	**3** ^**x**^ **10** ^**−7**^
**ATG12**	**0.42**	**5** ^**x**^ **10** ^**−4**^	**0.25**	**0.044**	**0.26**	**0.037**
**ATG14**	**0.51**	**2** ^**x**^ **10** ^**−5**^	**0.21**	**0.092**	**0.61**	**5** ^**x**^ **10** ^**−8**^

*Note*: Rho indicates Spearman correlation coefficient for DDR2 with the osteogenic signature RUNx2 (runt‐related transcription factor 2) and the autophagy markers ULK (autophagy activating kinase) 1, ATG (autophagy related) 9A, 10, 12, and 14. *N* = 64. Associations with *P* < 0.05 are marked in bold font.

### 3.4 Renal disease is significantly associated with DDR2 expression in thickened valve tissue

The patient characteristics of the 64 patients from which valves were obtained are presented in Table 3. Univariate correlations revealed significant negative correlations in healthy aortic valve tissue with statin use and in thickened aortic valve tissue with both BMI and renal disease. However, only the P‐value for the association with renal disease achieved the Bonferroni‐corrected statistical significance threshold (*P* < 0.003) imposed to adjust for multiple testing (Table 3). In the 9% of the patients that had renal disease, DDR2 expression was already decreased in the thickened aortic valve tissue to similar levels observed in the calcified tissue of patients with normal renal function (Figure 3c). Furthermore, VICs treated with high phosphate, one of the characteristics of CKD, revealed a phosphate‐induced inhibition of DDR2 expression (Figure S2).

**TABLE 3:** Patient characteristics and univariate correlations for DDR2 expression after stratification into different tissue characteristics of the aortic valve

		Healthy (*N* = 64)		Thickened (*N* = 64)		Calcified (*N* = 64)	
		Rho	*P*	Rho	*P*	Rho	*P*
Age, years	74.6 (72.0–77.3)	0.019	0.88	−0.070	0.58	−0.12	0.33
Sex, *N* (%) females	16 (25)	0.020	0.88	0.019	0.88	**0.27**	**0.029**
Smoking, *N* (%)		0.16	0.21	0.044	0.73	**0.30**	**0.016**
Current	4 (6)						
Former	33 (52)						
Never	27 (42)						
BMI, kg·m^−2^	26.9 (25.9–28.6)	0.072	0.60	**0.28**	**0.026**	0.17	0.19
Comorbidities							
CVD, *N* (%)	50 (78)	0.045	0.72	−0.11	0.38	−0.095	0.45
Diabetes, *N* (%)	14 (22)	−0.036	0.78	0.026	0.84	−0.12	0.36
Renal disease, *N* (%)	6 (9)	−0.068	0.59	**−0.40**	**0.0013**	−0.16	0.20
Medical treatments							
Aspirin	35 (55)	−0.22	0.079	−0.16	0.20	−0.048	0.70
Beta‐blockers	34 (53)	−0.053	0.67	−0.048	0.71	0.046	0.72
ACEi/ARB	18 (28)	−0.038	0.77	−0.22	0.075	−0.0019	0.92
Calcium channel blockers	22 (34)	−0.053	0.67	−0.070	0.58	0.038	0.76
Statins	39 (61)	**−0.26**	**0.040**	−0.20	0.12	−0.12	0.34

*Note*: Data are expressed as either median (95 CI) or N (%). Rho indicates spearman correlation coefficient. Renal disease was defined as either an estimated glomerular filtration rate less than 60 ml/min/1.73 m^2^ or the diagnosis of renal disease by International Classification of Diseases (ICD)‐10 codes N18.3, N18.4 or N18.3. BMI: body mass index; CVD: prevalent cardiovascular disease; ACEi: Angiotensin converting enzyme inhibitors; ARB: angiotensin receptor blockers. Associations with *P* < 0.05 are marked in bold font.

### 3.5 DDR2 is inhibited by nilotinib in human valvular interstitial cells

**FIGURE 4:**
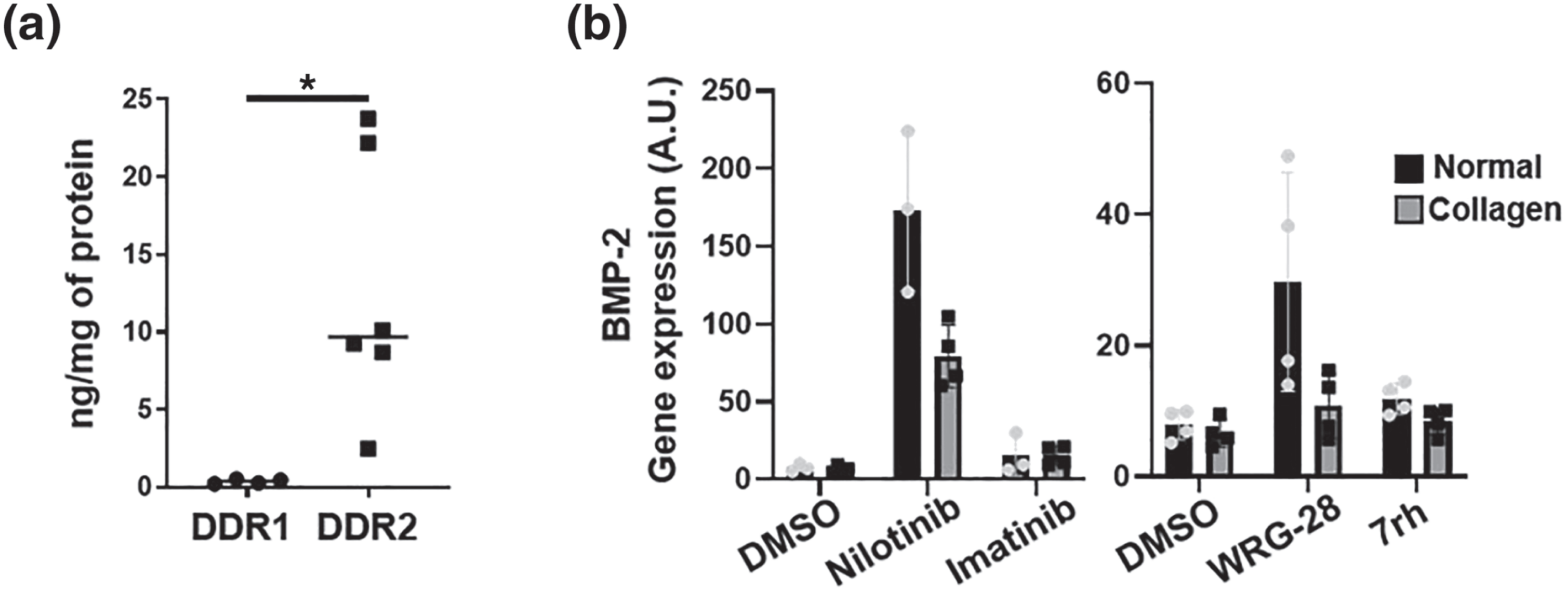
The tyrosine kinase DDR2 is preferentially expressed in human valvular interstitial cells and its inhibition leads to osteogenic activation. (a) DDR1 and DDR2 protein in human valvular interstitial cells (VICs) after 24 h in culture. *N* = 6 (DDR1 was undetectable in two samples). (b) BMP‐2 mRNA expression of VICs plated in polystyrene or collagen I‐coated plates treated for 24 h with control (DMSO), Nilotinib (10 μM), Imatinib (10 μM), WRG‐28 (10 μM) or 7rh (1 μM). Each dot represents one patient VIC donor *n* ≥ 3. Statistical significance of differences between groups were determined when at least *n* = 5 (a). *P < 0.05 for Student's *t* test

Isolation of VICs from human aortic valves revealed 34‐fold higher levels of DDR2 compared with DDR1 (Figure 4a), with further increased DDR2 expression over time in culture but decreased expression in response to high phosphate exposure (Figure S2). Neither of the two TKIs altered DDR2 protein expression levels in VICs (Figure S2). In preliminary findings, BMP‐2 mRNA levels were increased in response to nilotinib and the selective DDR2 inhibitor WRG28, whereas neither imatinib nor the selective DDR1 inhibitor 7rh altered BMP2 expression (Figure 4b). Stimulating DDR activation by culturing VICs on a collagen matrix (Day et al., 2008) blunted both the nilotinib‐ and WRG28‐upregulation of BMP‐2 (Figure 4b). Comparing effects on VIC calcification in an additional set of experiments (using VICs from an additional *n* = 6 patients) confirmed that nilotinib (10 μM) induced a 1.33 ± 0.29‐fold increase in calcification and showed similar effects of WRG28 (10 μM), which induced a 1.25 ± 0.81‐fold increase in calcification (Figure S3A). In contrast, the 0.47 ± 0.08‐fold decrease in VIC viability was not observed in WRG28‐treated cells (fold change 1.06 ± 0.91) (Figure S3B).

## 4 DISCUSSION

In the present study, nilotinib arises as a potential risk factor for accelerating the development of CAVS, based on a multitude of observations. First, nilotinib treatment increased aortic valve thickness in mice. Second, nilotinib inhibited the tyrosine kinase DDR2, which was highly expressed in human aortic valves and VICs, and its expression was downregulated with calcification. Third, nilotinib acted as an inducer of calcification, promoting osteogenic signalling and inhibiting autophagy. Taken together, these results implicate DDR2‐inhibition by nilotinib in aortic valve calcification, with direct translational implications for cardio‐oncology.

Aortic valve thickening in hyperlipidaemic mice represents a model of early valvular changes with similar characteristics to human disease in terms of osteogenic activation (Aikawa et al., 2007) and valvular iron (Laguna‐Fernandez et al., 2016; van Broekhoven et al., 2019). The present study revealed that nilotinib, but not imatinib, significantly increased murine valve thickness in hyperlipidaemic APOE*3Leiden.CETP transgenic mice that have a humanized lipoprotein metabolism and exhibit aortic valve thickening at a young age (van Broekhoven et al., 2019), which represents a model of early valvular changes with similar characteristics to human CAVS disease in terms of osteogenic activation (Aikawa et al., 2007), detected through BMP2 stainings in the present study, and valvular iron (Laguna‐Fernandez et al., 2016; van Broekhoven et al., 2019). Interestingly, nilotinib‐treated mice also exhibited a higher proportion of valvular iron positivity, which has been shown to precede calcification in human stenotic aortic valves (Morvan et al., 2019). Taken together, this model reinforces the role of iron accumulation and valve thickening as early markers of murine valve disease despite the lack of positivity for either Alizarin Red or von Kossa (Aikawa et al., 2007). Moreover, these differential findings between imatinib and nilotinib are in line with observations in CML patients, in which the incidence of myocardial infarction was approximately 3.6‐fold higher among patients treated with nilotinib compared with those treated with imatinib (Dahlen et al., 2016), and with the increased susceptibility to coagulation after nilotinib‐treatment in mice (Pouwer et al., 2018). However, the reason for these differential cardiovascular effects between first and second generation TKI, and the pathways linking nilotinib to valve disease have not previously been investigated.

We recently reported that nilotinib, but not imatinib, significantly increased calcification and mRNA levels of the osteogenic activator BMP‐2 in human VICs (Carracedo et al., 2020). Here, we confirm these finding using a different VIC calcification model and investigating BMP‐2 protein levels, and extend the observation by showing preliminary findings that nilotinib increased the expression of Runx2, an osteoblast signature gene. Runx2 and BMP‐2, signalling through pSMAD 1/5/8, have been shown to drive valvular calcification in mice in vivo and in VICs in vitro (Gomez‐Stallons et al., 2016). Taken together with the differential effects on murine valve thickness, nilotinib, but not imatinib, emerged as a strong inducer of human VIC calcification. One mechanism contributing to the anti‐leukaemic effects of nilotinib is through altered autophagy. Autophagic flux may serve as a protective mechanism in aortic valves by counteracting osteogenic activation and calcification in human VICs (Carracedo, Persson, et al., 2019). In the present study, nilotinib significantly inhibited autophagic activity in VICs, accompanied by a similar degree of reduced VIC viability. Nilotinib is a known autophagy modulator (Kumar et al., 2021), but the exact mechanisms involved are largely unexplored. The present results indicate that nilotinib‐targeted pathways contribute to preserving valvular homeostatic autophagy, which is a protective mechanism in CAVS (Carracedo, Persson, et al., 2019).

To determine the specific targets for nilotinib in the aortic valve, a transcriptomic nilotinib target screen was performed in human aortic valves, comparing calcified and noncalcified tissue. Mapping the valvular expression levels of all tyrosine kinases susceptible to be inhibited by nilotinib, our analysis identified DDR2 as the second most expressed tyrosine kinase in the aortic valve, which has concomitantly been reported to be the second most inhibited kinase by nilotinib (Manley et al., 2010). DDR2 was in addition one of the three nilotinib‐targets that were significantly down‐regulated in calcified compared with healthy human aortic valve tissue.

In the patient cohort used for the target screen, 9% of the patients had renal disease. Since even relatively mild CKD increases the risk of CAVS (Vavilis et al., 2019) and since valvular calcification is highly prevalent in CKD patients (Rattazzi et al., 2013), it is noteworthy that DDR2 expression decreased at an earlier stage of disease in valves from patients with CKD compared with normal renal function. Furthermore, DDR2 expression was decreased by high phosphate treatment of VICs. The decrease in calcification inhibitors is a key feature of VIC and aortic valve calcification (Bäck et al., 2018), which appears prematurely in CKD (Ebert et al., 2020). Hence, the present results point to pathways involving DDR2 as an additional protective mechanism against valve calcification. Pharmacological DDR1 and DDR2 targeting revealed that only DDR2 inhibition mimicked the response to nilotinib in terms of BMP2 induction and calcification in VICs, further supporting DDR2 inhibition by nilotinib as of importance for its pro‐calcifying effects. In contrast, VIC viability was not affected by selective DDR2 inhibition, suggesting that nilotinib affects additional valvular pathophysiological pathways.

Extracellular matrix remodelling and calcification are two of the hallmarks of AVS, which lead to the characteristic thickening of the aortic valve. The DDR2 tyrosine kinase is one of two discoidin domain nonintegrin receptors for collagen (Day et al., 2008), mediating cell to ECM interactions and implicated in cell migration, proliferation, differentiation, and survival. DDR1 expression in vascular smooth muscle cells (VSMCs) (Leitinger, 2014) regulates collagen remodelling (Ferri et al., 2004), fibrosis, and calcification (Krohn et al., 2016; Ngai et al., 2020). While those studies suggested that DDRs play a role in soft tissue calcification, their role in valvular heart disease has not previously been explored. Compared with DDR2 however, DDR1 exhibited low valvular expression and a significant increase in thickened and calcified valve tissue as well as in VICs. Whereas nilotinib and imatinib similarly inhibited DDR1, only nilotinib significantly inhibited DDR2 phosphorylation in the present study, which is in accordance with previous work performed in cell‐free TR‐FRET kinase assays (Day et al., 2008).

Since collagen inhibits VIC calcification, and its disruption promotes VIC apoptosis (Rodriguez et al., 2014), an intact collagen sensing by means of DDR2 may be instrumental for preserving homeostatic VIC function. In preliminary findings, stimulating DDR activation by culturing VICs on a collagen matrix (Day et al., 2008) blunted both the upregulation of BMP‐2 induced by both nilotinib and the selective DDR2 inhibition. These results provide a suggestion that enhancing DDR2 activity by means of increasing its collagen ligand blunted the BMP2‐induction observed after DDR2 inhibition. In further support of DDR2 being a possible link between nilotinib and VIC responses, DDR2 expression was inversely associated with the osteoblast signature RUNx2 and positively associated with autophagy markers.

In summary, nilotinib increased aortic valve thickness in mice and enhanced calcification, promoted osteogenic activation, and inhibited autophagy in human VICs. Further, we found DDR2‐inhibition by nilotinib and that DDR2 was highly expressed in human aortic valves and VICs, with a downregulated expression pattern with calcification. Valvular DDR2 expression was associated with osteogenic and autophagy pathways. The nilotinib‐induced osteogenic activation and calcification of VICs were reproduced by selective pharmacological DDR2 targeting. Taken together, the direct valvular effects of nilotinib point to DDR‐2 as part of the link between TKI and CAVS, although additional nilotinib‐effects may be exerted in aortic valves.

Although concerns for valvular adverse effects should not limit anti‐leukaemic treatment in CML (Carracedo et al., 2020; Steegmann et al., 2016), an awareness of CAVS may lead to earlier diagnosis and treatment of valve disease in CML patients. Identifying TKI‐targeted tyrosine kinases that contribute to cardiovascular homeostasis may open up personalized medicine with specific TKI profiles in CML.

## AUTHOR CONTRIBUTION

Conceptualization, A.F.‐C., E.E., L.S., and H.M.G.P, and M.B.; Performed experiments: M.C., S‐C.P., G.A., M.G.P, O.P., P.S‐G., and E.P. Writing original draft, M.C. and M.B.; writing—review and editing, all authors.

## CONFLICT OF INTEREST

The authors declare no conflict of interest.

## DECLARATION OF TRANSPARENCY AND SCIENTIFIC RIGOUR

This Declaration acknowledges that this paper adheres to the principles for transparent reporting and scientific rigour of preclinical research as stated in the *BJP* guidelines for Design and Analysis, Immunoblotting and Immunochemistry, and Animal Experimentation, and as recommended by funding agencies, publishers and other organizations engaged with supporting research.

## Supporting information


**Figure S1.** **Immunostaining of murine aortic roots for bone morphogenetic protein BMP‐2.** Representative images of sections from n = 3 mice from each group are shown for the controls in upper panels and nilotinib‐treated mice in the bottom panels.
Figure S2. **DDR expression and phosphorylation (A)** DDR1 and DDR2 fold change from vehicle control (dotted line) protein expression between healthy and calcified tissue in human aortic valves. **(B)** mRNA expression of DDR1 and DDR2 genes in human VICs in culture under calcifying conditions. **(C)** DDR1 phosphorylation level relative to vehicle control (dotted line) in VICs after 30‐minute stimulation with nilotinib (10 μM) or imatinib (10 μM). **(D)** DDR2 phosphorylation level relative to vehicle control (dotted line) in VICs after 30‐minute stimulation with nilotinib (10 μM) or imatinib (10 μM). **(E)** DDR2 protein level in VICs after 30‐minute stimulation with nilotinib (10 μM) or imatinib (10 μM). Data represented as mean ± S.D. Statistical significance of differences between groups was determined when at least n = 5 (A, D, and E). *P < .05,
**Figure S3. Effect of WRG‐28 on calcification and viability A)** Quantification of phosphate‐induced calcification of human valvular interstitial cells (VICs). **B)** VIC viability after treatment with nilotinib or WRG‐28. Data represented as mean ± S.D. N = 6 *P < .05.

## Data Availability

Data available within the article and supporting information.
