# Supplementary material for: The tyrosine kinase inhibitor nilotinib targets the discoidin domain receptor DDR2 in calcific aortic valve stenosis
Source: Br J Pharmacol. 2022 Jul 19;179(19):4709–21. doi: 10.1111/bph.15911 (PMC9544120; doi:10.1111/bph.15911)
Supplement: Supplementary file 1 — Figure S1. Immunostaining of murine aortic roots for bone morphogenetic protein BMP‐2. Representative images of sections from n = 3 mice from each group are shown for the controls in upper panels and nilotinib‐treated mice in the bottom panels. Figure S2. DDR expression and phosphorylation (A) DDR1 and DDR2 fold change from vehicle control (dotted line) protein expression between healthy and calcified tissue in human aortic valves. (B) mRNA expression of DDR1 and DDR2 genes in human VICs in culture under calcifying conditions. (C) DDR1 phosphorylation level relative to vehicle control (dotted line) in VICs after 30‐minute stimulation with nilotinib (10 μM) or imatinib (10 μM). (D) DDR2 phosphorylation level relative to vehicle control (dotted line) in VICs after 30‐minute stimulation with nilotinib (10 μM) or imatinib (10 μM). (E) DDR2 protein level in VICs after 30‐minute stimulation with nilotinib (10 μM) or imatinib (10 μM). Data represented as mean ± S.D. Statistical significance of differences between groups was determined when at least n = 5 (A, D, and E). *P < .05, Figure S3. Effect of WRG‐28 on calcification and viability A) Quantification of phosphate‐induced calcification of human valvular interstitial cells (VICs). B) VIC viability after treatment with nilotinib or WRG‐28. Data represented as mean ± S.D. N = 6 *P < .05. [file BPH-179-4709-s001.pdf]

**Figure S1**

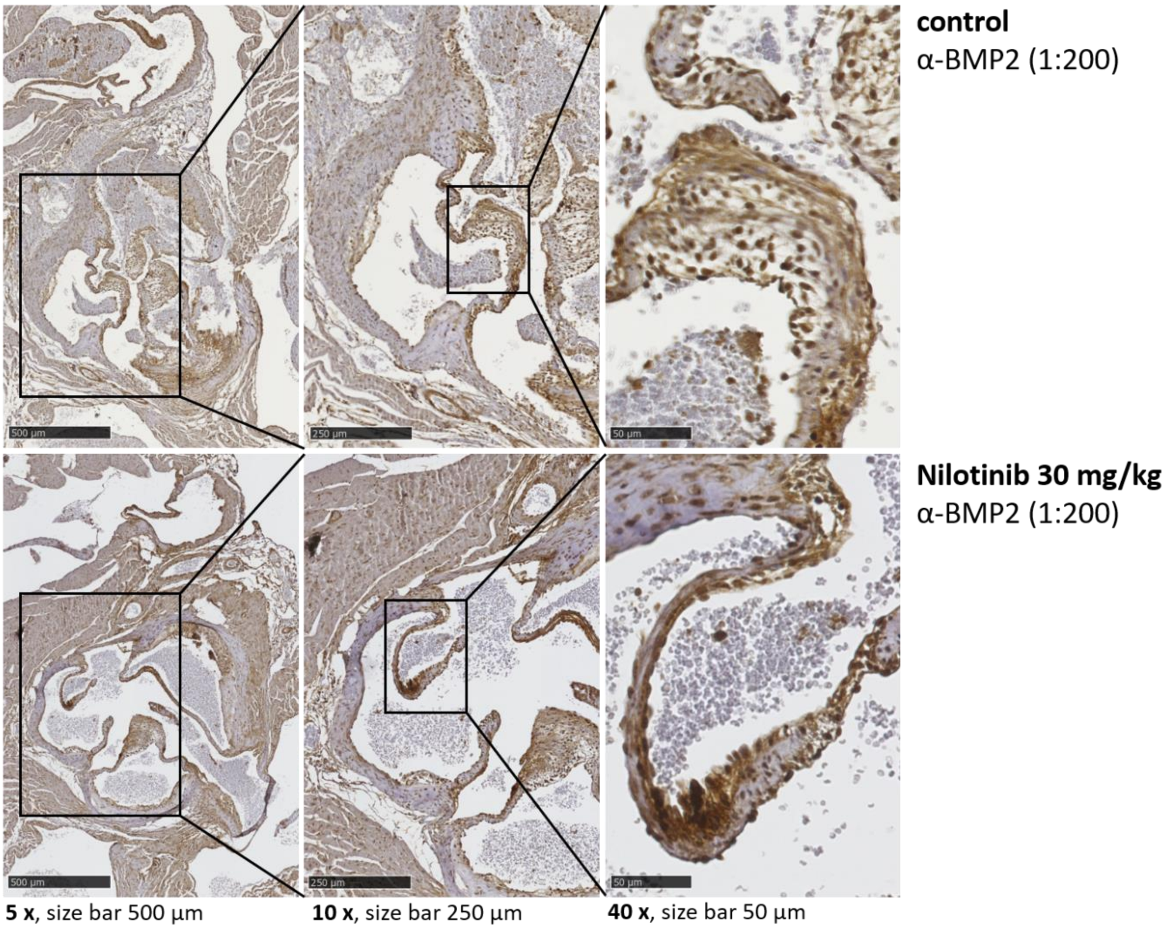


**Figure S1. Immunostaining of murine aortic roots for bone morphogenetic protein (BMP) 2.** Representative images of sections from n=3 mice from each group are shown for the controls in upper panels and nilotinib-treated mice in the bottom panels.

**Figure S2**

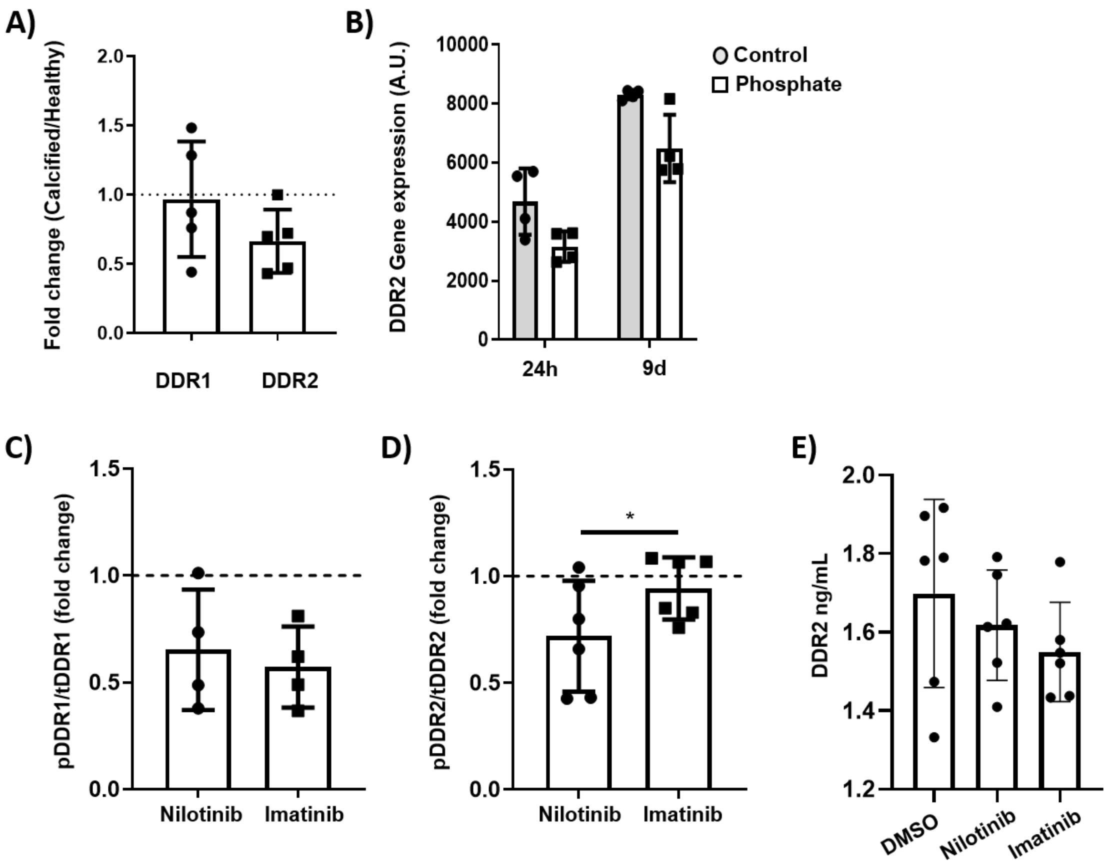


**Figure S2. DDR expression and phosphorylation (A)** DDR1 and DDR2 fold change to vehicle control (dotted line) protein expression between healthy and calcified tissue in human aortic valves. **(B)** mRNA expression of DDR1 and DDR2 genes in human VICs in culture under calcifying conditions. **(C)** DDR1 phosphorylation level to vehicle control (dotted line) in VICs after 30-minute stimulation with nilotinib (10 µM) or imatinib (10 µM). **(D)** DDR2 phosphorylation level to vehicle control (dotted line) in VICs after 30-minute stimulation with nilotinib (10 µM) or imatinib (10 µM). **(E)** DDR2 protein level in VICs after 30-minute stimulation with nilotinib (10 µM) or imatinib (10 µM). Data represented as mean ± S.D. Statistical significance of differences between groups were determined when at least n=5 (A, D, and E). *$P$<0.05,

**Figure S3**

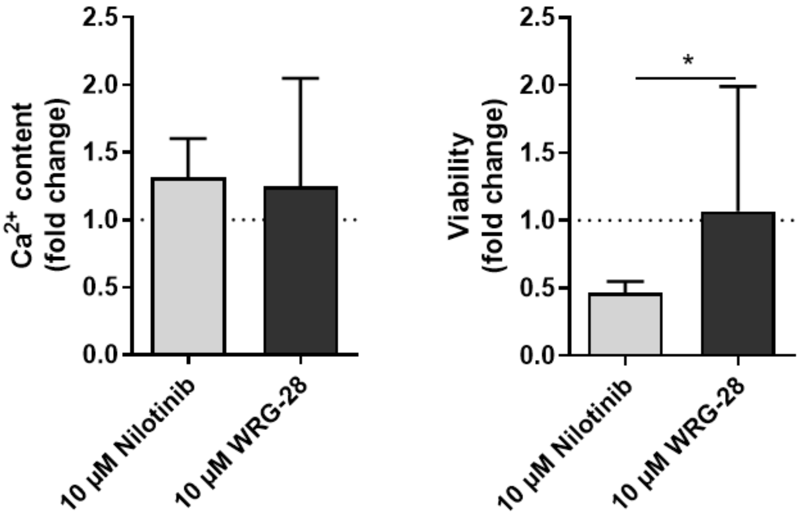


**Figure S3: Effect of WRG-28 on calcification and viability A)** Quantification of phosphate-induced calcification of human valvular interstitial cells (VICs). **B)** VIC viability after treatment with nilotinib or WRG-28. Data represented as mean ± S.D. N=6 *$P<0.05$.
